# Mechanical Effect of Performance Pressure Boots on Cadaveric Equine Hindlimb Fetlock Biomechanics

**DOI:** 10.3390/ani11040958

**Published:** 2021-03-30

**Authors:** Jennifer Symons

**Affiliations:** Shiley School of Engineering, University of Portland, Portland, OR 97203, USA; symons@up.edu; Tel.: +1-503-943-7435

**Keywords:** horse, metatarsophalangeal joint, suspensory ligament, jumping, pressure boots, pinch, flick

## Abstract

**Simple Summary:**

Pressure boots are performance enhancing equipment used by showjumping horses. Showjumping is scored by knocked down obstacle rails and time. Similar to weighted boots, pressure boots are intended to improve the hindlimb retraction of jumping horses to reduce the likelihood of knocking down rails on course. Manufacturers describe pressure boots as using acupressure to improve a horse’s awareness of their own limbs. However, this mechanism has not been verified within the scientific literature. The size and shape of features on the interior boot surface suggest a mechanical mechanism may affect anatomical structures within the lower limb. This research aims to characterize the mechanical effect of pressure boots by measuring forces and joint angles of cadaveric limbs with and without a pressure boot applied. Cadaveric limbs with a pressure boot applied required greater compressive loads to flex the fetlock joint than limbs without a pressure boot applied. This difference in compressive loads increased with increasing fetlock flexion angle. Differences in limb compressive loads contributed to greater tensile loads of palmar tendons and ligaments, specifically the suspensory apparatus. Greater tensile loading of tendons and ligaments may increase the likelihood of musculoskeletal injury and warrant concern for animal welfare of equine showjumping athletes.

**Abstract:**

Pressure boots are applied to hind limbs of showjumping horses with the intent to enhance jumping form. Manufacturers claim acupressure points enhance proprioception of hind limbs. With this increased awareness, horses are expected to retract their hind limbs to clear jump rails. This research aimed to investigate a more direct, mechanical effect of pressure boots on hind limb biomechanics. Cadaveric hind limbs (*n* = 6) were mechanically loaded in axial compression (3 cycles at 0.25 Hz, displacement control ~3300 N) with (2 trials) and without (2 trials) a pressure boot applied. During mechanical loading, fetlock angle was measured using bone fixed pins with retroreflective markers (30 Hz). Changes in limb load and fetlock angle between unloaded and loaded states, as well as average fetlock joint stiffness, were compared between trials with and without the pressure boot via ANOVA. Differences in measured loads between trials with and without the boot were observed in both unloaded (Δ = 6 N, *p* = 0.05) and loaded states (Δ = 25 N, *p* = 0.002). Trials with the boot had greater average fetlock stiffness (Δ = 3 N/degree, *p* = 0.001). Differences in loads with and without boots may increase with greater fetlock angles when cantering and jumping. These mechanical effects of pressure boots may contribute to greater tensile loading of palmar tendons and ligaments, and likelihood of musculoskeletal injury that can be related to animal welfare issues.

## 1. Introduction

Equine athletes in many disciplines undergo rigorous training. This training is necessary to maximize horses’ potential to perform well in competition. Horse owners, riders, and trainers implement many different strategies to maximize performance in equine athletes. Strategies may include nutrition, stabling environment, veterinary treatments, physiotherapy, massage, chiropractic work, acupuncture, training practices, arena surface management, shoeing, and equipment/tack choices.

Within showjumping, the design of distal limb coverings has been used to influence equine athlete performance, particularly hindlimb boots. Past studies have demonstrated the ability of boots to alter equine limb biomechanics. Several different types of limb coverings have been shown to decrease the amount of fetlock dorsiflexion during forelimb loading [1,2]. Additionally, weighted boots have been shown to alter the speed and degree of hindlimb retraction [3,4]. 

Prior research findings have supported the implementation of several rules regulating the design and use of distal limb coverings in equestrian competition. Within the past several years, both the FEI (Fédération Équestre Internationale) and United States Equestrian Federation (USEF) have implemented rule changes narrowing hind boot designs permitted in showjumping competitions [5,6]. These changes outline boot weight, dimensions, interior surface, strap compliance, and fastener types. Within the FEI, these rules were first applied to all horse and rider age-restricted classes. USEF hind boot rules were only applied to horse age-restricted classes, specifically young jumper classes (ages 5–8). The FEI recently extended these rules to all classes [5]. However, USEF presently has no reported plans to extend these rules to additional classes.

Several years ago, pressure boots were designed as an alternative to weighted boots that had been banned. These performance enhancing boots anecdotally produce similar increased limb retraction to clear obstacles in showjumping. Pressure boots incorporate protrusions on the medial and lateral interior surfaces of each boot. Manufacturers describe these protrusions as pushing on acupressure points intended to improve proprioception of the limb. Unfortunately, no prior published research has verified this proposed neural mechanism. 

Similar to weighted boots, pressure boots are not permitted within FEI and USEF rules governing open, rider and/or horse age-restricted classes. However, pressure boots are permitted in open classes within USEF, whereas weighted boots are not permitted in open classes within either governing bodies. Rule changes related to hind boots have received mixed support from riders and trainers globally. The gradual implementation of hind boot rules within competition brings into question whether public opinion within the industry will influence these rules being broadened or narrowed in the future. 

The use of performance enhancing equipment within equestrian sports spurs concerns of both unfair advantage in competition and animal welfare associated with possible musculoskeletal injury of strained tendons and ligaments during training and competition. Existing rules that restrict performance enhancing boots in horse and rider age-restricted classes may address both concerns in competition, but do not address the possibility of animal welfare issues during training. While the competitive advantage of performance enhancing equipment may be debated by riders in open classes, the lack of restrictions in some open classes fails to universally protect the welfare of all equine athletes. 

The size and placement of protrusions on the interior surface of pressure boots may affect palmar fetlock musculoskeletal structures, like the suspensory apparatus. This study aimed to investigate the mechanical effect of pressure boots on fetlock biomechanics in the absence of neural influences, including nociception and proprioception. Pressure boots were hypothesized to alter joint stiffness as it pertains to fetlock dorsiflexion and loading of palmar elastic energy storing tendons and ligaments. Specifically, limbs with a pressure boot applied were expected to have greater fetlock stiffness and require greater compressive loads to flex the fetlock joint compared to limbs without a pressure boot applied. Changes in fetlock stiffness and preferential increased loading of musculoskeletal structures may affect risk of injury, which has the potential to negatively impact animal welfare.

## 2. Materials and Methods

Hindlimbs from cadavers of 6 horses were used in this study. Known horse ages ranged from 2 to 6 years (Table 1). Limbs were provided through a donation program that accepts racehorses and pleasure riding horses after euthanasia. Horses had no diagnosed hindlimb musculoskeletal injuries when euthanized. No gross structural defects were observed in any study limbs. Racehorses donated typically range in age from 2 to 8 years, whereas pleasure horses donated are of adult to senior age. While study limbs differed in age and/or size compared to limbs of typical showjumping horses, all study limbs had normal, intact, distal limb anatomical structures of interest in this study. Limb sidedness (left or right) was assigned randomly to each horse, while ensuring an equal number of each limb side.

Each cadaveric limb was dissected of all muscles proximal to the point of the hock. Dissection of these muscles altered the proximal attachments of the superficial and deep digital flexor tendons that cross the fetlock. The lack of muscle activations and disruption of proximal attachment sites altered fetlock stiffness in cadavers compared to live horses. However, these alterations were uniform throughout all subsequent mechanical tests with and without a pressure boot applied. 

Each tibia was cut approximately 11 cm proximal to the point of the hock to facilitate casting of the distal tibia in polymethyl methacrylate using a cylindrical mold. Spherical motion capture markers (*n* = 14, 13 mm diameter) were mounted to each limb via a series of bone mounted fixation pins (*n* = 8). Transcortical mediolateral pins were placed at the proximal aspect of the third metatarsal bone, as well as the first and second phalanges. Lateral pins were placed at the distal aspect of the third metatarsal bone and first phalanx, as well as the hoof. Dorsal pins were placed at the distal aspect of the second phalanx and hoof. Pin and marker placement relative to anatomical structures were recorded via dorsopalmar and lateral radiographs with a calibration marker (70 kVp, 2 mAs). Protrusions on the interior surface of the pressure boots were positioned 6 cm proximal from a depression intended to wrap around the point of the fetlock (i.e., ergot or tuft of hair). Distance between the junction of the suspensory ligament and suspensory branches, and the point of the fetlock was measured to relate boot morphology (i.e., protrusion size and location) to limb anatomy.

**Figure 1:**
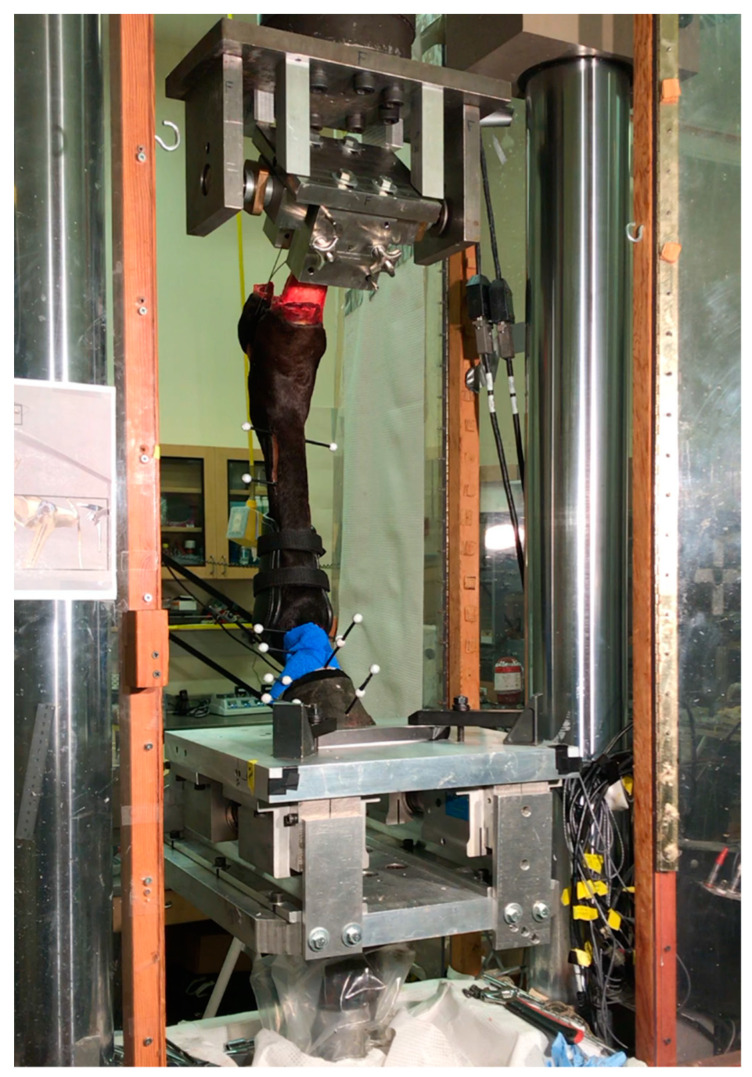
Cadaveric limbs were transected at the middle of the tibia and mounted proximally in a material testing system. The distal steel platform allowed for craniocaudal translation during limb loading. Motion capture markers were mounted to bone fixed pins in the third metatarsal bone, first and second phalanges, as well as the hoof.

Each cadaveric limb was mounted proximally in a material testing system (MTS) using a custom clamping fixture (Figure 1). The fixture angle (i.e., tibial orientation) was 38° caudal from vertical, which was informed by previous published kinematics data [7] and institutional lab knowledge of prior mechanical testing setups. To fix the hock angle in the absence of palmar muscle tendon units, the calcaneus was tethered to the proximal fixture. Skin and superficial soft tissues were removed from the medial and lateral aspects of the calcaneus to drill a mediolateral hole through this bone. Aircraft cable was then threaded through this bone hole, as well as through a hole in the proximal fixture, and tightened until the third metatarsal bone was roughly 5° caudal from vertical. This initial caudal position was needed to allow for some settling during preconditioning that would achieve vertical orientation prior to mechanical testing trials.

Distally within the MTS, the hoof was placed directly onto a steel platform that was able to translate cranially and caudally during limb loading. A toe block was mounted in front of the hoof to prevent translation of the hoof relative to the platform. Initial compressive loading was controlled manually (i.e., not controlled by computer) up to 1800 N. Each limb was then preconditioned for 200 cycles at 0.25 Hz from approximately 500 N to 1800 N (displacement controlled by computer, Δd unique to each limb). After this preconditioning, each third metatarsal bone maintained a vertical orientation under all loads greater than 500 N. Compressive loading was then manually increased to 3300 N (approximate peak load at walk).

**Figure 2:**
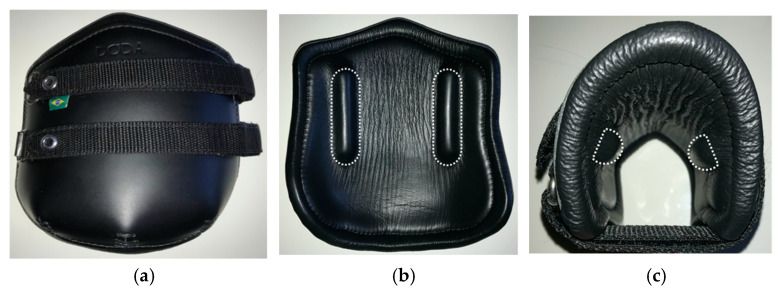
Pressure boot: (**a**) Boot exterior flat, (**b**) Boot interior flat, (**c**) Boot interior folded. Protrusions (1 cm × 1.5 cm × 8.5 cm, dotted outlines) were located roughly 8.5 cm from the distal edge of the boot and 3.5 cm palmar from the medial and lateral leading edges of the boot.

Each limb was tested for 4 trials, while alternating conditions with and without a pressure boot (Doda Jumping Boots Model I, Mikmar Bit Company, Divide, CO, USA; Figure 2) between each trial (2 trials with boot and 2 trials without boot). The pressure boot was applied manually by a single operator who was familiar with their use and appropriate strap tension on live horses. Each trial consisted of 3 cycles at 0.25 Hz from approximately 400 N to 3300 N (displacement controlled controlled by computer, Δd unique to each limb). Compressive load and stroke of the MTS, as well as three-dimensional motion capture video via two high-speed, high-resolution cameras (Fastcam PCI, Photron, Tokyo, Japan) oriented dorsomedially and dorsolaterally, were collected at 30 Hz.

Motion capture videos were processed using motion analysis software (Motus, Vicon Motion Systems, Oxford, UK). Discrepancies between pin/marker positions and bone longitudinal axes were accounted for by using virtual markers based on radiographic measurements. Fetlock joint angles were filtered using a 3 Hz lowpass, Butterworth filter. During the 3 cycles within each trial, 3 maximum loads were recorded, as well as 2 minimum loads. Limb loading and unloading were each observed twice within these maximum and minimum loads recorded during each trial. Typically, torsional stiffness of a joint is reported as the change in angle (°) in response to an applied torque (Nm). The testing setup allowed for an accurate measure of load applied to the limb, but did not allow for an accurate measure of changing moment arm during limb loading to calculate torque. Therefore, fetlock stiffness was reported as the ratio of change in load to change in fetlock angle during limb loading or unloading. Loads, joint angles, and fetlock stiffnesses were statistically compared (*p* = 0.05) between trials with and without a pressure boot applied using a one-way ANOVA (PROC MIXED, SAS Institute, Cary, NC, USA) to allow for repeated measures within each subject. Fixed effects included condition (with and without boot), as well as the interaction of condition and horse. Due to the small sample size, assumptions of the ANOVA were satisfied by visual assessment of residual distribution.

## 3. Results

Changes in fetlock angle and load during cadaveric distal hindlimb compression within a material testing system (MTS) were compared between trials with and trials without a pressure boot applied. Results from one cadaveric limb (Horse 1) were removed due to hoof translations with respect to the steel platform in the absence of a toe block. All reported results reflect the remaining five limbs.

No statistically significant differences were observed in fetlock angle between trials with and trials without the pressure boot (Table 2). However, statistically significant differences were observed in the load measured by the MTS. Trials with the pressure boot applied had greater compression resisting loads (+6 N, F = 4.27) in the unloaded condition (~400 N, minimum/starting load) compared to trials without the pressure boot. Trials with the pressure boot applied had greater differences in compression resisting loads (+25 N, F = 10.78) in the loaded condition (~3300 N, maximum load) compared to trials without the pressure boot.

Differences in compression resisting loads during unloaded and loaded conditions contributed to differences in fetlock stiffness metrics. Fetlock stiffness was calculated as the ratio of change in MTS load to change in fetlock angle during unloading (from ~3300 N to ~400 N) or loading (from ~400 N to ~3300 N). Trials with the pressure boot applied had statistically significantly greater fetlock stiffness (+3 N/°, F = 11.55) compared to trials without the pressure boot.

Pressure boot interior features interface with some suspensory apparatus structures. Measurements from the point of the fetlock to the junction of the suspensory ligament and branches ranged from 10 to 12 cm. Overlap between suspensory branches and protrusions ranged from 4 to 6 cm on the medial and lateral aspects of the limb. In most horses (except Horse 3), this measurement of overlap was strongly correlated (r = 0.88) with changes in fetlock stiffness between trials without and trials with the pressure boot applied. That is, larger measurements of overlap were concurrent with larger changes in fetlock stiffness when the pressure boot was applied.

## 4. Discussion

The mechanical effects of pressure boots were assessed by examining compression resisting loads and fetlock angles of cadaveric limbs with and without a pressure boot applied. Limbs with a pressure boot applied had greater compression resisting loads compared to limbs without a pressure boot applied. Furthermore, limbs with a pressure boot applied had greater fetlock stiffness, that is they required greater additional compressive loads to increase dorsiflexion of the fetlock joint. Therefore, the mechanical effect of pressure boots resists dorsiflexion of the fetlock. In the absence of limb coverings, palmar tendons and ligaments act to resist dorsiflexion of the fetlock. These findings may indicate manipulation and increased loading of palmar musculoskeletal structures by pressure boots that could negatively impact animal welfare.

The mechanical effects of pressure boots suggest involvement of palmar elastic energy storing structures. The fetlock joint primarily flexes dorsally and palmarly within the sagittal plane [8,9]. Therefore, any changes in fetlock joint stiffness may be attributed to dorsal and/or palmar loads that contribute to moments about the fetlock joint. These dorsal and palmar loads may be from external structures like leg coverings and/or internal structures like tendons and ligaments. Prior studies have investigated leg coverings with the ability to apply dorsal loads to the proximal phalanx and alter fetlock moments and range of motion [1,2]. However, pressure boots have open fronts that are not capable of applying dorsal loads to the pastern. Therefore, pressure boots are altering fetlock stiffness by influencing palmar tendons and/or ligaments of the fetlock.

In this experiment, fetlock biomechanics were primarily influenced by the passive suspensory apparatus. Cadaveric limbs in this study were transected at the middle of the tibia, so origins and muscle bodies of the superficial and deep digital flexor tendons were disrupted. These origins and muscle bodies will likely contribute to greater loads and fetlock stiffnesses in live horses. The deep digital flexor tendon has an accessory ligament tethered to the tarsus that gives this muscle-tendon unit some ligament properties. However, hindlimb accessory ligaments are substantially less developed than those in the forelimbs and hypothesized to contribute less in locomotion [10,11]. Therefore, accessory ligament contributions to fetlock biomechanics were present, but minimal. The suspensory apparatus was the only palmar fetlock support structure unaltered in cadaveric specimens, and thus is primarily responsible for any changes in observed fetlock stiffness with the application of pressure boots. However, these results do not fully capture how pressure boots may preferentially alter suspensory apparatus loading relative to palmar fetlock tendons in a live horse.

Non-elastic pressure boot straps may contribute to suspensory ligament strain and fetlock stiffness. A previous study observed no change in fetlock biomechanics over a wider range of motion (up to 70° dorsiflexion) when the limbs of live horses were covered by compliant bandages and neoprene boots that fully encompassed the cannon bone [1]. Conversely, pressure boots used in this experiment have nylon straps that minimally elongate when loaded in tension. This lack of compliance maintains a rigid circumference binding the suspensory ligament to the palmar aspect of the third metatarsal bone. This restriction causes the ligament to strain and bow, particularly during limb loading. Similar effects would be expected for the superficial and deep digital flexor tendons in a live horse. Furthermore, non-elastic boot straps with high stiffness are not exclusive to pressure boots and may be present in other types of fore and hind limb coverings, which pose similar concerns.

Protrusions on the interior surface of pressure boots may alter suspensory ligament strain and fetlock stiffness. When a pressure boot is applied to a horse’s distal limb, these protrusions are positioned adjacent to the medial and lateral branches of the suspensory apparatus. With the aid of non-elastic circumferential straps, pressure from these protrusions draws the medial and lateral suspensory branches axially toward each other. If the length of the suspensory apparatus is held constant at two fixed endpoints, this inward movement requires the branches to elongate. Elongation or strain within the branches transmits tensile loads to all proximal and distal structures in series. Therefore, pressure boot strain of suspensory branches can contribute indirectly to suspensory ligament strain. Furthermore, the magnitude of overlap between protrusions and suspensory branches appears to be related to the mechanical effect size of pressure boots on fetlock stiffness in some horses. Placement of protrusions to increase this overlap may increase the observed mechanical effects of pressure boots.

Prior equine biomechanics research may inform projections of loads during cantering and jumping with pressure boots. Due to the repeated measures of this study, tests were limited to in vivo walking loads. Under these conditions, fetlock angles reached 35° dorsiflexion. However, previous studies have observed up to 75° fetlock dorsiflexion in horses jumping 1.5 m [12]. This additional 40° of flexion, coupled with an estimated limb stiffness of 3 N/°, would contribute to 120 N added to the observed compression resisting loads measured by the MTS. Furthermore, prior analyses have verified that loads within palmar tendons and ligaments of the fetlock exceed ground reaction forces measured at the hoof during stance. A galloping racehorse, which has a similar magnitude of fetlock dorsiflexion as jumping horses, is expected to have suspensory ligament loads 1.5 times greater than ground reaction forces at the hoof [13]. Based on these previous estimates, showjumping horses could experience up to an additional 218 N applied to the suspensory ligament when jumping with pressure boots (i.e., 1.5 × [25 N + 40° × 3 N/°]). Suspensory ligament loads have been estimated to exceed 11 kN in galloping racehorses [13,14] and 15 kN in jumping horses without pressure boots applied [15]. These baseline estimates during galloping and jumping meet or exceed failure stresses of cadaveric suspensory ligaments [16] and likely contribute to plastic deformations of tendon and ligament fibers in live horses. Therefore, additional loads from pressure boots may alter limb loading and warrant concerns for risk of musculoskeletal injury, particularly when considering repetitive loading in showjumping training and competition.

The presence of neural mechanisms proposed by manufacturers may exaggerate the observed mechanical effects of pressure boots. Proprioception is most often physiologically assessed by sensory mechanoreceptors within tendons and ligaments that cross joints. These mechanoreceptors send signals to the central nervous system to inform voluntary and involuntary control strategies. For instance, the patellar reflex test in humans produces excitatory and inhibitory muscle signals in response to strains in the patellar tendon. Proprioceptive mechanisms in the equine distal limb are poorly understood but have been hypothesized to involve distal structures of the suspensory apparatus [17]. If similar excitatory reflexes exist in horses, strain in the suspensory apparatus to activation of palmar muscle tendon units like the superficial and deep digital flexor tendons could further contribute to increased resistance to fetlock dorsiflexion and enhance palmar flexion.

The findings of this research are limited by the use of a limited number of cadaver limbs and a single type of pressure boot. Cadaveric limbs are limited in examining any structures with active, neural components. Results represent passive musculoskeletal structures and do not fully capture biomechanics of live horses, particularly neural control and muscle activation. Additional studies investigating live horses are required to characterize these components. Furthermore, cadaveric limbs were obtained through postmortem donations. Therefore, the age and breed of donated limbs were based on availability. The age and breed of donated limbs differed from typical showjumping horses. Donated limbs were obtained from younger and/or smaller breed horses. Limbs from typical showjumping horses that are older and/or from larger breeds would be expected to have the same anatomical structures, but of larger size compared to measured limbs. These anatomical size differences may affect the magnitude of observed mechanical effects of pressure boots. Therefore, the observed limbs serve to establish the presence of a mechanical effect of pressure boots, but the magnitude of this effect will likely vary between individual horses based on anatomical differences in distal limbs. Additionally, a single type of pressure boot was investigated in this study. Many brands offer pressure boots that all share protrusions on the medial and lateral interior surfaces of boots. However, the size, shape, and location of these protrusions may differ between brands. The interaction of differences in limb and boot morphology may alter the magnitude of reported effects of pressure boots on limb biomechanics.

## 5. Conclusions

In the absence of neural mechanisms like nociception and proprioception, pressure boots mechanically effect musculoskeletal structures in cadaveric distal hindlimbs of horses, particularly the suspensory apparatus. High level showjumping contributes to high loads within the suspensory apparatus near failure loads. Additional loads applied to the suspensory apparatus by pressure boots may alter fetlock stiffness and limb loading, as well as influence risk of musculoskeletal injury. Organizations governing equine showjumping competitions should consider the mechanical effects of pressure boots on musculoskeletal structures when establishing rules to permit or not permit their usage. Similarly, owners, riders, and trainers should be educated about the mechanical effects of pressure boots and any potential negative impacts to musculoskeletal tissues.

## Figures and Tables

**Table 1:** Horse age, breed, cadaver limb side, and starting condition.

Horse	Age (years)	Breed	Hindlimb	Starting Condition
1	2–3	TB	L	Without boot
2	6	TB	R	Without boot
3	unknown	TB	R	Without boot
4	unknown	Pleasure	L	With boot
5	unknown	TB	L	With boot
6	unknown	Pleasure	R	With boot

**Table 2:** Statistical comparison of load within materials testing system (MTS), fetlock angle, and fetlock stiffness between trials with and without a pressure boot applied during loaded (3300 N) and unloaded (400 N) states. Fetlock angle is the dorsal angle between the third metatarsal bone and the first phalanx (i.e., angles less than 180° indicate dorsiflexion).

Variables (Loading Phase(s) Underlined)	Without Boot	With Boot	Difference	*p*-Value
Unloaded				
MTS load (N)	402 ± 2	408 ± 2	6 ± 3	0.05
Fetlock angle (°)	170 ± 0	170 ± 0	0 ± 0	0.08
Loaded				
MTS load (N)	3337 ± 5	3361 ± 5	25 ± 7	0.002
Fetlock angle (°)	145 ± 0	145 ± 0	0 ± 0	0.94
Unloading/Loading				
∆MTS load (N)	2934 ± 4	2951 ± 4	18 ± 5	0.002
∆Fetlock angle (°)	25 ± 0	25 ± 0	0 ± 0	0.03
Fetlock stiffness	121 ± 1	124 ± 1	3 ± 1	0.001

## Data Availability

Not applicable.
